# Study on Strengthening Mechanism of Arc Welding Interface Between TiC Steel-Bonded Carbides and Welding Material Based on First Principles

**DOI:** 10.3390/ma19132901

**Published:** 2026-07-06

**Authors:** Wei Wei, Shaokang Guan, Zhiquan Huang, Shuhao Zhu, Haiyan Zhang

**Affiliations:** 1 China Academy of Machinery Zhengzhou Research Institute of Mechanical Engineering Co., Ltd., Zhengzhou 450001, China; weiw@zrime.com.cn (W.W.); zrime7435275@163.com (Z.H.); 2 School of Material Science & Engineering, Zhengzhou University, Zhengzhou 450001, China; skguan@zzu.edu.cn; 3 Institute for Genetic Engineering of Materials, Henan Academy of Sciences, Zhengzhou 450046, China; 15534496624@163.com

**Keywords:** TiC steel-bonded carbides, arc welding, first principles, multi-component composite reinforcement, core–rim structure

## Abstract

In this study, the effects of W, Cr, and Mo alloying elements on the microstructure and mechanical properties of the arc welding interface between TiC steel-bonded carbide and welding material were investigated using TEM, SEM, microhardness testing, and mechanical property testing. First-principles calculations were employed for verification. The results indicate that, under the experimental conditions, the shear strength of the arc welding interface between the ZDZC60 carbide and the multi-component composite welding material (enhanced with W, Cr, and Mo) was 83% higher than that before optimization. The order of the three metal atoms when substituting Ti atoms in TiC particles was determined as W > Mo > Cr. The “core–rim” structure of TiC particles in the partially melted zone (PMZ) of the arc welding interface was proposed. The coherent structure of TiC particles, characterized by (200)TiC//(200)TiMoC_2_//(200)TiWC_2_ and [011]TiC//[011]TiMoC2//[011]TiWC2, was identified. W and Mo atoms exert an inhibitory effect on the dissolution of TiC particles, while Cr atoms disperse in the binder phase in the form of carbides and solid solutions, thereby effectively strengthening the interface.

## 1. Introduction

With the continuous improvement of the global industrial level and the rapid development of science and technology, the equipment manufacturing industry is gradually moving towards large-scale production, harsh working conditions, and high-efficiency operation. Large-scale equipment such as excavators and crushers constitutes an important part of the national manufacturing industry [1,2]. Key components like liners, grinding rollers, and grinding disks are core wear-resistant parts of excavators and crushers. These parts endure prolonged exposure to extreme service environments and experience severe material loss, resulting in significant annual consumption. Developing wear-resistant materials is a crucial research direction for extending the service life of wear-resistant parts in large-scale equipment and an important task for enhancing the overall competitiveness of the national manufacturing industry.

TiC steel-bonded carbide uses TiC or Ti(C,N) as the hard phase and alloy steel mainly composed of elements such as Ni, Mo, and Fe as the binder phase, which is manufactured by metallurgy. The emergence of TiC steel-bonded carbide fills the gap in comprehensive performance between tool steel, cemented carbide, and ceramic materials [3,4]. TiC steel-bonded carbides are extensively utilized in the manufacturing of wear-resistant components due to their higher hardness and higher wear resistance compared to steel matrix while also offering good machinability, heat treatability, and forgeability compared to conventional hard metals [5,6,7,8]. However, due to the limited size of TiC steel-bonded carbide products, they must be joined to steel matrix for application in large-scale equipment.

Feng [9] employed Zn-doped Ag-Cu composite filler metal to braze TiC cermet to steel substrate. The brazing process was conducted at 850 °C with a holding time of 10 min, and a sound metallurgical bonding interface was successfully obtained. A mutual dissolution zone formed between the Ag-Cu-Zn filler metal and TiC cermet at the interface, which consisted of binder phases derived from the filler metal and TiC particles. The maximum shear strength of the joint reached 120.7 MPa. Laansoo [10] joined TiC cermet via induction brazing using Ag449 filler metal. After post-heat treatment at 250 °C, the interfacial shear strength increased from approximately 250 MPa to 290 MPa. Li [11] adopted a Ti-Nb-Cu interlayer to realize vacuum diffusion bonding between TiC cermet and 304 stainless steel. Under the conditions of bonding temperature of 925 °C, holding time of 20 min and pressure of 8 MPa, the interfacial shear strength of the joint reached 84.6 MPa. Maurya [12] fabricated TiC-based cermets via pulsed selective laser melting (SLM). The results revealed that laser processing is an effective approach to suppress crack initiation. Nevertheless, process limitations lead to the formation of defects such as pores, which deteriorate the toughness of the material.

Arc welding is a widely used, highly adaptable, convenient, and effective joining method with simple operation [13]. However, arc welding has a large heat input, and the remelting of TiC particles during the welding process can degrade the interface structure and properties. There are few reports on the arc welding of TiC steel-bonded carbide to steel matrix, especially on the structure and properties of the weld interface. In particular, during the arc welding process of TiC steel-bonded carbides, challenges such as remelting, diffusion, and migration of TiC hard phases at the weld interface, as well as degradation of mechanical properties, arise, thereby restricting their application in wear-resistant components for large-scale equipment.

To address the critical need for high-performance joining of TiC steel-bonded carbides to steel matrix, it is necessary to consider not only the bonding at the arc welding interface between the weld metal and the carbide, but also the interface bonding between the binder phase and TiC hard phase under the influence of heat input. The poorly understood mechanism of microstructure transformation and performance evolution at the weld interface between TiC steel-bonded carbide and steel substrate has limited its application in wear-resistant parts of large-scale equipment.

In previous studies [14,15] the optimal welding heat input for ZDZC60-type TiC steel-bonded carbide was considered to be 6.2 kJ/cm, and the optimal arc welding material was low-carbon steel. The shear strength of the arc welding interface between low-carbon steel (LCS) and ZDZC60 is lower than that of both the as-sintered ZDZC60 and the ZDZC60 after thermal cycling. The arc welding interface is a weak link of the sample. However, the microstructure morphology can be controlled and the grain size can be refined by designing the composition of the deposited metal, thereby improving the strength and toughness of the arc welding interface [12,13]. For the alloy strengthening with W, Cr, and Mo, the optimal alloy ratio was determined as 1 wt.% W, 0.5 wt.% Cr, and 2 wt.% Mo.

Understanding how changes in welding material composition affect the microstructure and properties of the bonding phase, as well as the physicochemical characteristics of TiC particles, is key to researching the microstructure and performance of the ZDZC60 fusion welding interface. In this study, the TiC core–rim mechanism under W-, Cr-, and Mo-enhanced LCS arc welding thermal cycles and the mechanical response of the microstructure and mechanical properties of the arc welding interface between the W-, Cr-, and Mo-optimized LCS and ZDZC60 were analyzed. The substitution of W, Cr, and Mo atoms for Ti atoms in TiC was verified based on first principles via the spin-unrestricted Density Functional Theory (DFT) method.

## 2. Materials and Methods

ZDZC60 is a TiC steel-bonded carbide fabricated by powder metallurgy. In this material, TiC serves as the hard phase, while alloying elements including Ni, Mo, Mn and Fe act as the binder phase. The size of ZDZC60 is 60 mm × 26 mm × 20 mm. Quantitative chemical analysis was performed to examine the elemental composition, and the corresponding results are listed in Table 1.

**Figure 1:**
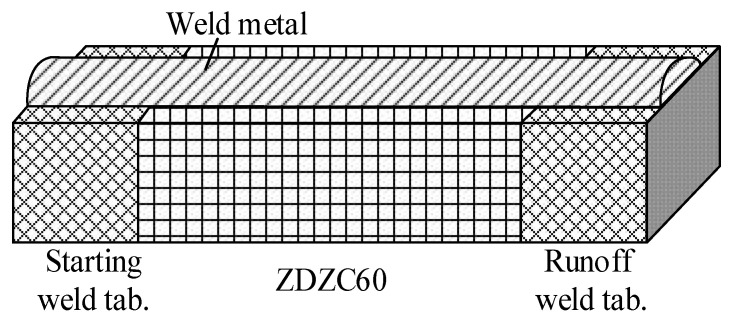
Exhibition of arc welding process.

One deposited sample was prepared according to the parameters in Table 2 [14]. The welding robot model M-10iA12 manufactured by FANUC (Shibokusa, Japan) was used in the experiments, combined with a digital welding power source of Pulse MIG-500RP type produced by AOTAI (Jinan, China). Neither preheating before welding nor post-weld heat treatment was applied, and all joints were cooled in air after single-pass single-layer welding. During arc welding, the ZDZC60 specimens were placed flat on the worktable. Given the small size of the samples, run-on and run-off tabs were arranged at both ends to avoid defects at the arc start and arc stop regions during specimen sampling, as shown in Figure 1. The deposited metal was prepared using a shielding gas mixture of 80% Ar and 20% CO_2_. Welding was performed by ϕ1.6 mm flux-cored wire, achieving a deposit thickness of 18–20 mm, composed of 5 layers, with a continues arc welding process. The chemical analysis results are shown in Table 3. The results show that the deposited metal contains 0.96 wt.% W, 0.55 wt.% Cr, and 1.94 wt.% Mo. These compositions meet the optimal mass fraction design requirements obtained from previous orthogonal experiments [15]. A 0.5 mm thick metal foil sample was cut from the specimen using molybdenum wire electrical discharge machining, mechanically pre-thinned to about 50 μm, and then placed in a GL-6960 ion beam thinning instrument for ion milling until a perforation at the sample center allowed light transmission. The microstructure at the interface was observed using an FEI Talos F200X field-emission TEM (Brno, Czech Republic).

The shear strength (*τ_bb_*) of the specimen was tested in accordance with GB/T 6400-2007 [16]. Impact energy and flexural strength (*σ_bb_*) were measured in accordance with GB/T 25774.1-2023 [17].

The spin-unrestricted Density Functional Theory (UDFT) method in the DMol3 code of the Materials Studio software package 2020 [18] was performed in this study to support the qualitative tendency of the substitution energy of W, Cr and Mo to substitute Ti in TiC particles. The UDFT was attempted regarding the high temperature during the welding process which might induce spin polarization. The electron exchange and correlation effects were described using the Generalized Gradient Approximation (GGA) based on Perdew–Burke–Ernzerhof (PBE), and the Double Numerical plus Polarization (DNP) basis set was adopted [19].

On-the-fly-generated (OTFG) ultrasoft pseudopotentials were employed to characterize the electron–ion interactions. All computational simulations were performed using the conventional unit cell. An orbital occupation smearing value of 0.005 Ha was adopted to optimize the electronic convergence efficiency. A 3 × 3 × 1 Monkhorst–Pack grid was utilized for Brillouin zone integration [20]. During geometry optimization, all atomic positions were fully relaxed with a maximum of 100 ionic relaxation steps and a maximum atomic step size of 0.3 Å. The unified convergence tolerances for total energy change, maximum atomic force, and maximum atomic displacement were set to 1.0 × 10^−5^ eV, 0.03 eV/Å, and 0.005 Å, respectively. The self-consistent field (SCF) convergence criterion was consistent with the geometric optimization tolerance. K-point density measurements were performed to guarantee the precision and reliability of all computational results.

## 3. Results

### 3.1. Mechanical Properties

**Figure 2:**
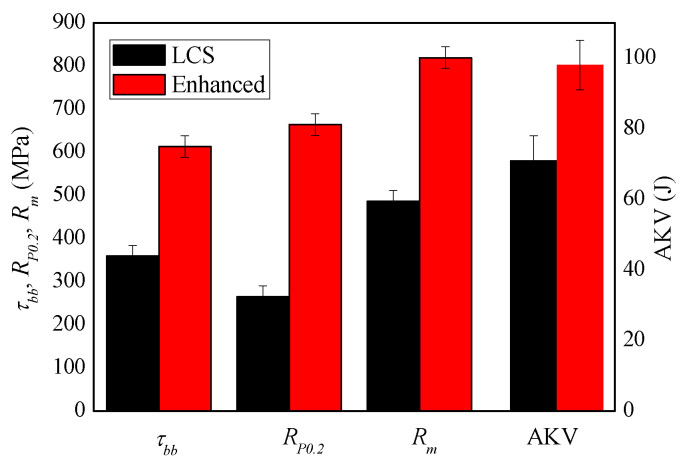
Comparison of shear strength between deposit of multi-component composite-enhanced LCS and LCS.

The average mechanical properties of the deposited metal are summarized in Table 4, which includes results from one tensile specimen, three impact specimens, and three shear specimens. A comparison of the mechanical properties of the low-carbon steel (LCS) deposited metal before and after multi-component composite reinforcement is presented in Figure 2.

Table 4 and Figure 2 show that the mechanical properties of the multi-component composite-reinforced LCS deposited metal are significantly superior to those of the original LCS deposited metal. Specifically, the shear strength increased from 359 MPa to 613 MPa (a 70.7% increase), the yield strength (*R_p_*_0.2_) from 265 MPa to 664 MPa (a 150.5% increase), the tensile strength (*R_m_*) from 486 MPa to 819 MPa (a 68.5% increase), and the 0 °C impact energy (AKV) from 71.7 J to 98.0 J (a 36.7% increase). These results demonstrate that under the present experimental conditions, the multi-component composite reinforcement achieves an evident enhancement in overall mechanical properties of the deposit.

**Figure 3:**
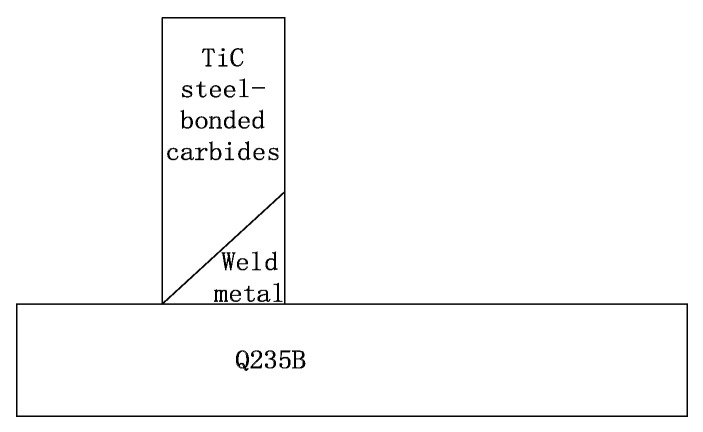
Mechanical property tests preparation.

Mechanical property tests were conducted on the joint between ZDZC60 and a Q235B steel substrate, with the joint configuration shown in Figure 3. The base metal was Q235B, and a single-side V-groove T-joint with a butt weld (45° groove angle) was used.

The shear strength was tested according to GB/T 6400-2007. The test results are presented in Table 5. Table 5 shows that the average shear strength of the arc welding interface between the multi-component composite-reinforced LCS weld metal and ZDZC60 was 797 MPa. This value is 83% higher than the shear strength of a ZDZC60 thermal simulation sample under a heat input of 6.2 kJ/cm at 430 MPa [10]. The joint exhibited an average bending strength of 903 MPa and an average 0 °C impact energy of 3.5 J. Fracture occurred on the ZDZC60 side of the joint. Since ZDZC60 itself has low impact toughness, the joint’s impact energy is comparable to that of the ZDZC60 base metal.

**Figure 4:**
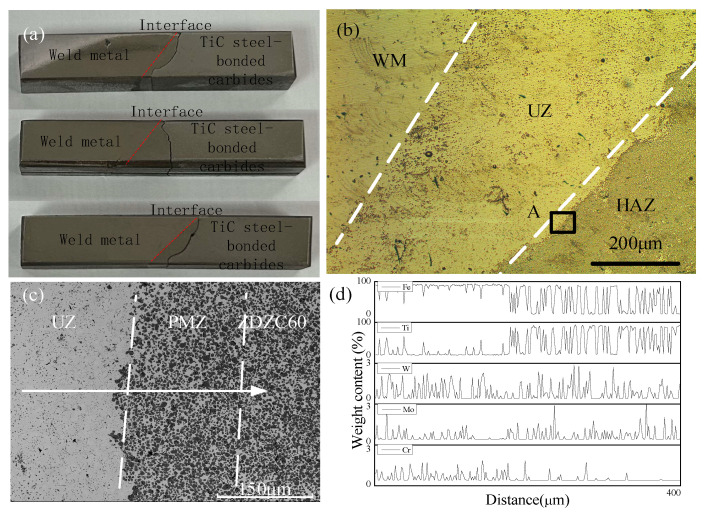
Macroscopic morphology, OM image, SEM and EDS result of interface of impact test specimen: (**a**) macroscopic morphology after impact test; (**b**) OM image of arc welding interface; (**c**) SEM result of arc welding interface; (**d**) EDS line scan results of line in (**c**).

The impact test samples of the joint were observed and analyzed, and the results are shown in Figure 4. Figure 4a shows the macroscopic morphology of the sample after the impact test, where the left side of the fracture was the weld metal and the right side was the TiC steel-bonded carbide (ZDZC60). All fracture positions occurred on the ZDZC60 side. The microstructure of the interface was observed, with typical characteristics presented in Figure 4b. SEM observation was performed on the boxed area A in Figure 4b to analyze the interface bonding between ZDZC60 and the weld metal, as shown in Figure 4c.

Figure 4c reveals that the right side of the arc welding interface was the ZDZC60 heat-affected zone (HAZ) with densely distributed TiC particles. The middle part was the partially melted zone (PMZ), which also contains densely distributed TiC particles, but the binder phase was ferrite. The left side was the unmixed zone (UZ). According to the definition, the UZ forms during cooling as in situ-formed TiC particles combine with the bonding phase and weld metal. This zone features a ferrite matrix with a small number of in situ-generated TiC particles (1–3 μm in size) dispersed within it. Figure 4d shows the results of linear scanning energy dispersive spectroscopy (EDS) along the white line marked in Figure 4c. The EDS results indicate the diffusion of alloying elements at the ZDZC60 arc welding interface within the PMZ, confirming good metallurgical bonding between the weld metal and ZDZC60. Specifically, the concentrations of Fe and Cr gradually decrease from the weld metal towards the ZDZC60 side, while the Ti concentration shows a gradual increasing trend. The distributions of W and Mo elements are uniform on both sides of the arc welding interface.

**Figure 5:**
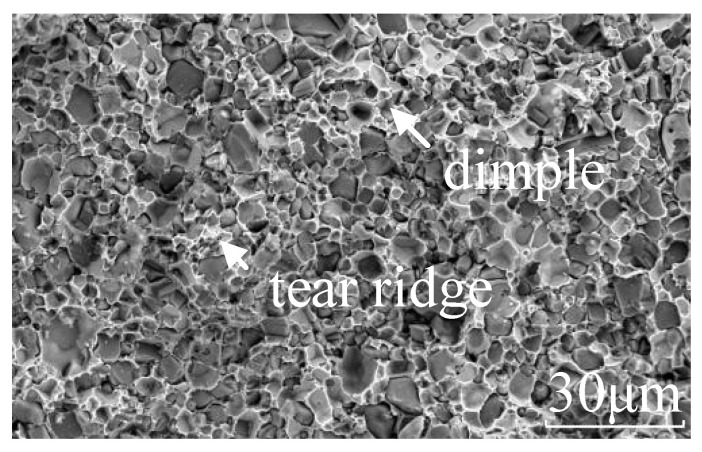
Shear fracture morphology of arc welding interface.

The microstructure and morphology of the impact fracture at the ZDZC60 arc welding interface were observed, as shown in Figure 5. Obvious secondary cracks in TiC particles, tear ridges and dimples in the binder phase can be observed on all shear fracture surfaces of the specimens. This indicates that the shear fractures of all three groups exhibit a ductile–brittle mixed fracture characteristic, which is consistent with the typical feature of quasi-cleavage fracture. The fracture surface microstructure does not exhibit the ductile fracture characteristics of the weld metal and is consistent with the fracture mode of TiC steel-bonded carbide. Therefore, the fracture location is on the TiC steel-bonded carbide side.

### 3.2. First Principles

**Figure 6:**
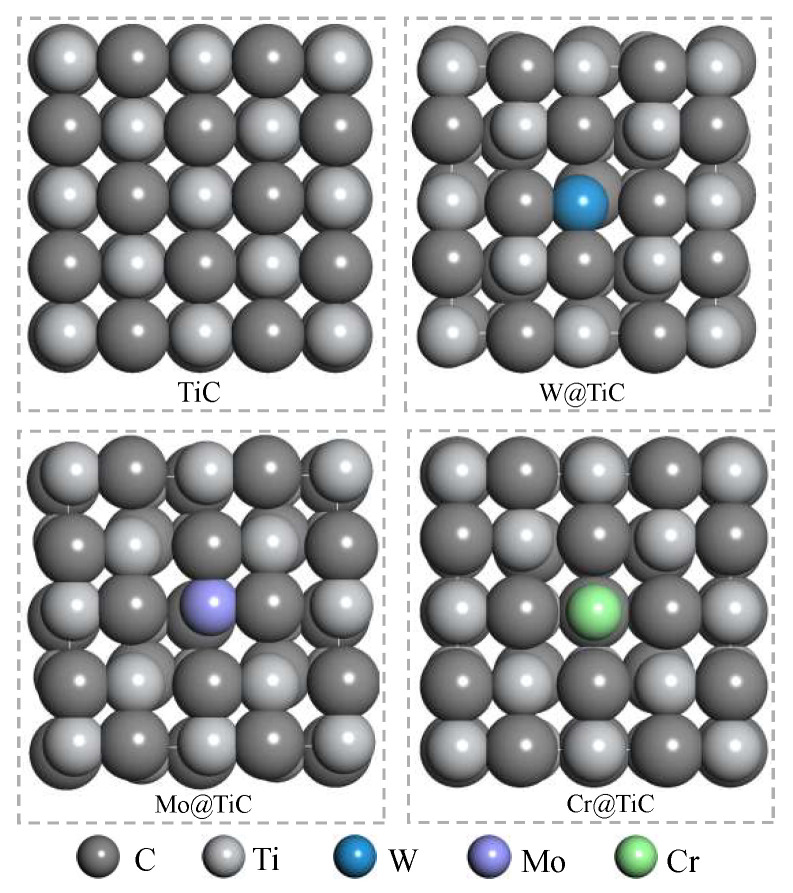
Exhibition of W, Cr, and Mo atoms substituting Ti atoms in TiC.

Figure 6 shows the constructed and optimized structures of TiC and M-TiC. Analysis of Figure 6 shows the face-centered cubic (FCC) structure has almost no distortion, indicating that the structure remains stable after W, Cr, and Mo atoms substitute for Ti atoms in TiC, and stable compounds can be formed.

The average bond length (d_ave_) of M-C after substitution was calculated, and it was obvious that all d_ave_ values were close to 2 Å. The d_ave_ values of the three M-C bonds were slightly different, which may be caused by the differences in atomic radius and electromagnetism of W, Cr, and Mo metal atoms. To further evaluate the deviation of metal atoms in M-TiC, the deviation degree (ε) of metal atoms can be calculated using Formula (1):

(1) $$\varepsilon =\left[1-\frac{d_{M-C\left(\min\right)}}{d_{M-C\left(\max\right)}}\right]\times 100\%$$

where d_M-C(min)_ is the minimum value of the M-C bond length, and d_M-C(max)_ is the maximum value of the M-C bond length.

The calculation results of ε are shown in Table 6. After W atoms substituted for Ti atoms, there was almost no deviation from the center of M-TiC (ε < 10%), indicating that W-TiC has good symmetry. After Mo and Cr atoms substituted for Ti atoms, the deviation degree was relatively high (ε > 20%), resulting in poor stability. The order of the three metal atoms substituting Ti atoms in TiC was W > Mo > Cr.

The substitution energy (E_d_) required for W, Cr, and Mo atoms to substitute for Ti atoms in TiC was calculated, as shown in Formula (2):

(2) $$E_{d}=E_{Metal@TiC}-E_{Metal}+E_{Ti}-E_{TiC}$$

where E_Metal@TiC_ is the lattice energy after atomic substitution, E_Metal_ is the energy of the substituted metal atom, E_Ti_ is the energy of a single Ti atom, and E_TiC_ is the lattice energy of TiC.

**Figure 7:**
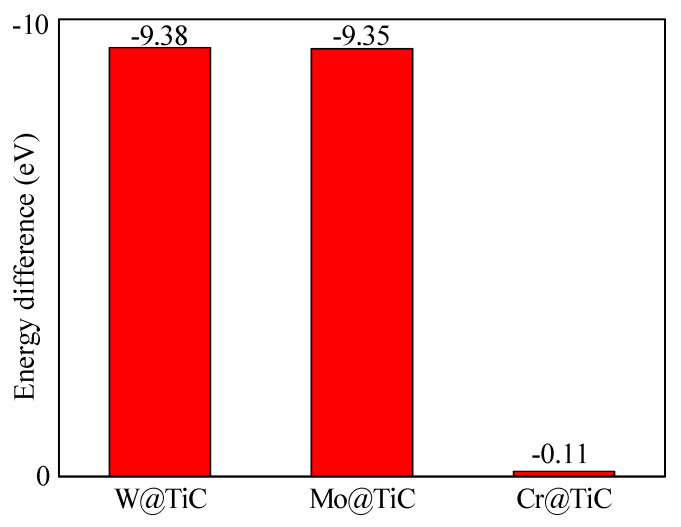
E_d_ with W, Cr, and Mo atoms replacing Ti atoms in TiC.

**Figure 8:**
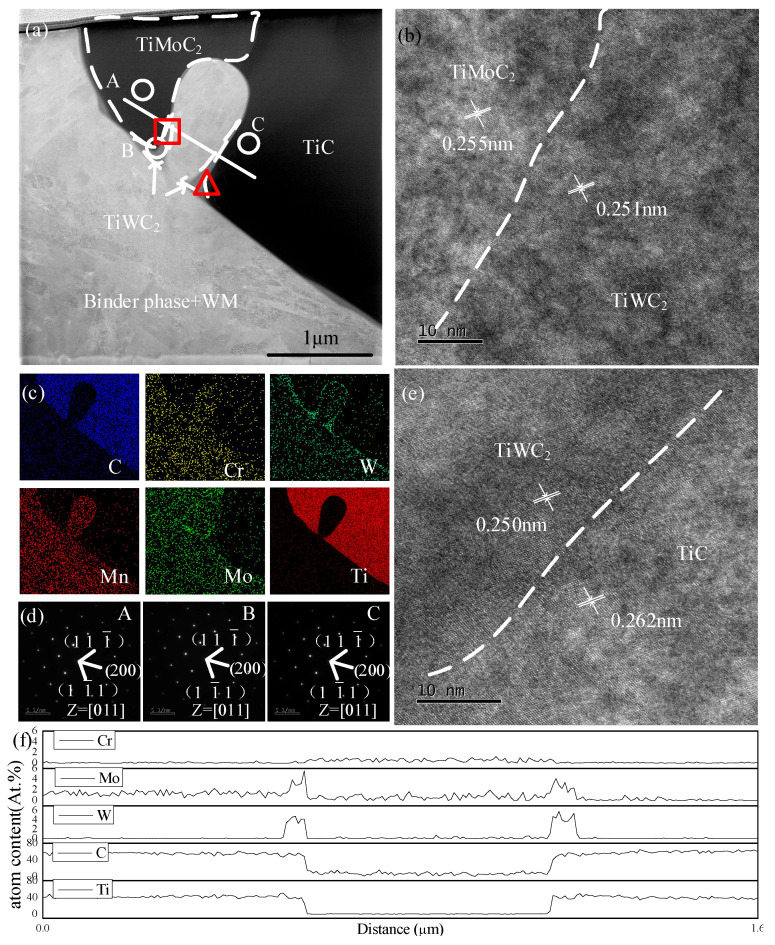
TEM images of TiC particles in arc welding interface between multi-component composite-enhanced LCS and ZDZC60: (**a**) TEM image; (**b**) HRTEM of the square site in (**a**); (**c**) EDS results of (**a**); (**d**) diffraction patterns at circles site in (**a**); (**e**) HRTEM of the triangle site in (**a**); (**f**) EDS line scan results of the white line in (**a**).

A negative calculated value of E_d_ means that substituting Ti with other metal atoms was energetically favorable. Furthermore, the more negative the E_d_ value, the easier it is to substitute for Ti atoms. The calculation results of E_d_ values used for qualitative comparison under the selected computational reference are shown in Figure 7. It can be seen from Figure 7 that all E_d_ values were negative, indicating that the substitution of Ti atoms in TiC by W, Cr, and Mo atoms was favorable, and the M-TiC structures can all achieve thermodynamically stable structures. In addition, by comparing the E_d_ values of the three metal elements, it can be found that W element has the smallest E_d_ value. Under the high-temperature effect of arc welding, W atoms substitute Mo atoms in the outer ring of sintered TiC particles and form a stable TiWC_2_ structure with TiC. Since arc welding is a process of rapid heating and rapid cooling, the reaction time was short. Meanwhile, Cr atoms have the largest E_d_ value among the three atoms, so they can hardly undergo substitution reactions with TiC in the molten pool. From the EPMA element distribution results of TiC particles at the arc welding interface in Figure 8, it can be seen that TiC particles form a three-layer structure with TiC as the core, TiMoC_2_ in the middle, and TiWC_2_ as the outer ring. Cr was dispersed in the matrix without an obvious annular structure.

### 3.3. Microstructure

The microstructure analysis results are presented in Figure 8. Figure 8a shows a TEM image of TiC particles observed from the PMZ. The boxed area in Figure 8a was further examined by high-resolution TEM (HRTEM) as shown in Figure 8b, and the triangular area is shown in HRTEM in Figure 8e. EDS mapping analysis of the region in Figure 8a is presented in Figure 8c. Selected-area electron diffraction (SAED) patterns from circular areas A, B, and C in Figure 8a are shown in Figure 8d, and linear EDS scanning results along the white line in Figure 8a are displayed in Figure 8f. Figure 8a,c show that the left side corresponds to the iron-based binder phase, where Mn and Cr alloying elements are dispersed. The dark area on the right side consists of TiC particles. Within these particles, Ti, Mo, and C elements are concentrated in the upper half, identified as TiMoC_2_ by XRD [11]. The lower half, primarily composed of Ti and C, forms the “core” of the TiC particles. A W-enriched layer approximately 0.1 μm wide exists between the TiC core and the binder phase, identified as TiWC_2_ by XRD [21]. No Cr segregation was observed in the maps, indicating that Cr does not react with TiC but remains dissolved in the matrix. Lattice coherency at the interfaces was assessed by HRTEM. At the TiMoC_2_/TiWC_2_ interface [Figure 8b], the interplanar spacings are 0.255 nm and 0.251 nm, respectively, yielding a lattice mismatch of less than 2%. Similarly, at the TiWC_2_/TiC interface [Figure 8e], the spacings are 0.262 nm and 0.250 nm, with a mismatch below 5%. These small mismatches allow elastic strain to accommodate the lattice differences without introducing defects, indicating a stable composite lattice for the annular structure formed by TiC, TiWC_2_, and TiMoC_2_. The SAED patterns in Figure 8d confirm that TiMoC_2_ (area A), TiWC_2_ (area B), and TiC (area C) all share a face-centered cubic (FCC) structure with identical crystallographic orientation. The orientation relationships are (200)TiC//(200)TiMoC_2_//(200)TiWC_2_ and [011]TiC//[011]TiMoC2//[011]TiWC2, demonstrating a fully coherent structure among the three phases. The linear scan profile in Figure 8f further supports this microstructure. Cr was absent in the regions corresponding to TiMoC_2_, TiWC_2_, and TiC, being primarily located in the binder phase, confirming its non-participation in the annular structure formation. The TiC core region contains only Ti and C, while W and Mo are concentrated in the TiWC_2_ and TiMoC_2_ regions, respectively. Together, TiC, TiWC_2_, and TiMoC_2_ constitute a coherent “core–shell” structure for the TiC particles in the partially melted zone (PMZ).

## 4. Discussion

According to the “core–rim” theory [22], TiC particles partially dissolve under the arc heat input during welding, releasing Ti and C atoms into the mixed weld metal and ZDZC60 binder phase. During subsequent cooling, W—having the lowest substitution energy among the alloying elements (W, Mo, Cr)—preferentially substitutes into the carbide lattice. This leads to the initial formation of TiWC_2_ on the outer surface of the pre-existing TiMoC_2_ layer on the TiC particles. Ultimately, a coherent TiC-TiMoC_2_-TiWC_2_ annular structure develops. Concurrently, Cr atoms, which do not readily incorporate into this carbide structure, precipitate at grain boundaries or form fine inclusions within the matrix.

Microstructure analysis of the arc-welded interface confirms that TiC particles in the partially melted zone (PMZ) indeed possess this three-layer (TiC core, TiMoC_2_ and TiWC_2_ rim) annular structure. This stable structure effectively inhibits the complete melting and decomposition of TiC particles under the experimental conditions. Instead of forming a carbide rim, Cr contributes to strengthening by forming fine Fe-Cr-C solid solutions dispersed in the matrix.

It was noteworthy that the present structure (TiC-TiMoC_2_-TiWC_2_) differs from the TiC-Ti(W,Mo)C-Ti(Cr,Ni)C annular structure reported by Jin [23]. This discrepancy was attributed to the distinct processing conditions. Jin employed liquid-phase sintering with a prolonged high-temperature dwell time (up to 240 min), allowing sufficient diffusion for even Cr (despite its higher substitution energy) to form an outer Ti(Cr,Ni)C rim. In contrast, arc welding involves rapid thermal cycles with a short liquid-phase duration. Within this limited time, only W and Mo, with their favorable kinetics, can diffuse to form the outer rim layers (TiWC_2_ and TiMoC_2_), resulting in a double-layer rim structure. Orthogonal experiment analysis further indicates that a small amount of finely dispersed Cr-rich inclusions can beneficially enhance the overall shear strength.

During arc welding, PMZ occurs on TiC steel-bonded carbide, resulting in different reaction zones at the interface. Considering the complex nonequilibrium melting, rapid atomic diffusion and transient high-temperature solidification features of actual welding, the first-principles simulation is only focused on clarifying the substitution effect of W, Cr and Mo atoms on TiC particles under instantaneous high-temperature reaction conditions.

The formation mechanism described above highlights the critical role of rapid thermal cycling in shaping the unique double-layer rim structure. While this structure has successfully improved mechanical properties under laboratory conditions, practical application in complex service environments remains a challenge. Therefore, further simulation experiments under realistic service conditions are warranted to evaluate the long-term bonding strength and reliability of the arc-welded ZDZC60 interface. Manufacturing advanced wear-resistant parts with ZDZC60 can extend their service life in large-scale equipment, an important task for enhancing the overall competitiveness of the national manufacturing industry.

## 5. Conclusions

Through orthogonal experiments, the proportions of W, Cr, and Mo alloying elements in the LCS welding material were optimized, leading to the development of a welding material suitable for arc welding of ZDZC60. The strengthening and toughening mechanisms of the arc-welded interface were elucidated, with the main conclusions as follows:

(1) The results of the microstructure and properties of the arc-welded interface between the multi-component composite-enhanced LCS welding material and ZDZC60 indicate that the optimal interface structure and performance were achieved when the mass fractions of W, Cr, and Mo were 1.0 wt.%, 0.5 wt.%, and 2.0 wt.%, respectively.
(2) Under the experimental conditions, the shear strength of the arc-welded interface using the self-developed W-Cr-Mo multi-component composite-enhanced LCS welding material and ZDZC60 was 83% higher than that before optimization, which significantly improved the bonding strength. Concurrently, the mechanical properties of the weld metal itself were enhanced.
(3) A “core–rim” annular structure was identified for TiC particles within the partially melted zone (PMZ) of the ZDZC60 arc-welded interface. First-principles calculations show that W and Mo atoms, substituting for Ti in TiC, tend to segregate to the particle periphery. This leads to the formation of a double-layer rim structure with a TiC core surrounded by successive layers of TiMoC_2_ and TiWC_2_. The mechanisms responsible for the enhanced interface strength were clarified: the W- and Mo-rich rim inhibits the dissolution of TiC particles; Cr atoms contribute solid-solution strengthening in the binder phase; and finely dispersed nano carbides provide additional dispersion strengthening.

## Figures and Tables

**Table 1:** Chemical composition of ZDZC60 (wt.%).

C	Mn	Ni	Mo	Ti	Fe
8.2~8.4	6.0~6.3	1.7~1.8	1.3~1.4	33.0~35.0	46.5~47.5

**Table 2:** Arc welding process parameters.

*I* (A)	*U* (V)	*v* (cm/s)	(L/min)
270	27	0.8	15–20

**Table 3:** Components of deposit of multi-component composite-enhanced LCS (wt.%).

	W	Cr	Mo	C	Si	Ni	Fe
Enhanced LCS	0.96	0.55	1.94	0.04	0.21	0.35	Rem.
LCS	/	/	0.50	0.05	/	0.44	Rem.

**Table 4:** Mechanical properties of deposit of multi-component composite-enhanced LCS.

	*τ_bb_* (MPa)	*R_p_*_0.2_ (MPa)	*R_m_* (MPa)	0 °C AKV (J)
Enhanced LCS	613	664	819	98.0
LCS	359	265	486	71.7

**Table 5:** Mechanical properties of arc welding joint of ZDZC60 and Q235b.

Specimen ID	*τ_bb_* (MPa)	*σ_bb_* (MPa)	0 °C AKV (J)
1#	797	894	3.7
2#	810	912	3.3
3#	783	903	3.6

**Table 6:** *d_ave_* (Å) with W, Cr, and Mo atoms replacing Ti atoms in TiC.

	TiC	W@TiC	Mo@TiC	Cr@TiC
*d_ave_* (Å)	2.166	2.201	2.212	2.283
*ε*	0	6.436	22.760	25.523

## Data Availability

The original contributions presented in this study are included in the article. Further inquiries can be directed to the corresponding author.

## Abbreviations

The following abbreviations are used in this manuscript:

LCS	Low-Carbon Steel
WM	Weld Metal
PMZ	Partially Melted Zone
UZ	Unmixed Zone
